# Bacterial Pathogens of Bovine Mastitis: Prevalence, Antimicrobial Susceptibility, and Sensitivity to *Caesalpinia sappan* Both In Vitro and In Vivo Studies

**DOI:** 10.3390/biology14040350

**Published:** 2025-03-27

**Authors:** Phacharaporn Tadee, Wiwat Pattanawong, Apichart Manwicha, Pakasinee Khaodang, Doungporn Amornlerdpison, Sunee Chansakaow, Pramote Tipduangta, Kridda Chukiatsiri, Pakpoom Tadee

**Affiliations:** 1 Faculty of Animal Science and Technology, Maejo University, Chiang Mai 50290, Thailand; phacharaporn.boonkhot@gmail.com (P.T.); wpattanawong@gmail.com (W.P.); apichart.m@hotmail.com (A.M.); pakasinee.pk@gmail.com (P.K.); 2 Center of Excellence in Agricultural Innovation for Graduate Entrepreneur, Maejo University, Chiang Mai 50290, Thailand; doungpornfishtech@gmail.com; 3 Faculty of Fisheries Technology and Aquatic Resources, Maejo University, Chiang Mai 50290, Thailand; 4 Faculty of Pharmacy, Chiang Mai University, Chiang Mai 50200, Thailand; sunee.c@cmu.ac.th (S.C.); phiptpdn@gmail.com (P.T.); 5 Faculty of Veterinary Medicine, Chiang Mai University, Chiang Mai 50100, Thailand

**Keywords:** mastitis, antimicrobial resistance, herbal extract, *Caesalpinia sappan*, treatment, disease control

## Abstract

Mastitis is a major infectious disease that causes significant economic losses in the dairy industry. Current control programs primarily rely on antimicrobials, contributing to the growing concerns of the resistance situation and drug residues in milk. This study aimed to identify the bacterial pathogens responsible for subclinical/clinical bovine mastitis, assess their antimicrobial resistance profiles, and evaluate the antimicrobial effects of *Caesalpinia sappan* in treating mastitis through both in vitro and in vivo studies. A total of 138 bacterial isolates, representing 40 species, were identified from 100 milk samples collected from dairy cows under the Maejo Cooperative Group in Chiang Mai, Thailand, between May 2021 and February 2022. The most prevalent species was *Escherichia coli* (10.87%), followed by *Bacillus cereus* (9.42%) and *Staphylococcus sciuri* (7.97%). The highest resistance rates were observed for penicillin (65.21%), followed by streptomycin and lincomycin (61.59%). Nine isolates resisted all 18 antimicrobials tested. The average minimum inhibitory concentration (MIC) of *C. sappan* against the identified pathogens ranged from 0.63 to 17.68 mg/mL, with the highest MIC observed against *Pseudomonas luteola*. In the animal experiment, treatment with a prototype of an intramammary infusion compound primarily formulated from *C. sappan* significantly reduced the total bacterial count and California Mastitis Test (CMT) scores (*p* < 0.01). These results suggest that *C. sappan* exhibits antimicrobial efficacy against various mastitis bacteria and could serve as a potential alternative treatment to control bovine mastitis in dairy cattle within the study region.

## Simple Summary

Current bovine mastitis control programs primarily rely on antimicrobial use, raising concerns about antimicrobial resistance and drug residues. Herbal medicine presents a promising substitute. In this study, several antimicrobial-resistant mastitis-causing pathogens were identified, most of which exhibited susceptibility to *Caesalpinia sappan* extracts. In an animal experiment, treatment with a prototype intramammary infusion primarily formulated from *C. sappan* significantly reduced disease severity. These findings suggest that this medicinal plant holds potential as an alternative treatment for bovine mastitis.

## 1. Introduction

Bovine mastitis, an inflammatory condition of the mammary gland and udder tissue, is one of the most significant economic concerns in the dairy industry [1]. This condition leads to increased treatment costs, discarded milk, reduced milk yield and quality, higher culling/replacement costs, and negative impacts animal welfare [2,3]. The disease can be classified according to clinical features (clinical vs. subclinical) and etiology (non-infectious vs. infectious). The infectious causes are most common; bacteria are the most prevalent. *Staphylococcus aureus*, *Streptococcus agalactiae*, *Streptococcus dysgalactiae*, *Streptococcus uberis*, *Klebsilla pneumoniae*, and *Escherichia coli* are the most frequently identified [4]. Intramammary administration of antimicrobials is a well-established method for eliminating causative bacteria from infected quarters [5]. However, this approach is not always effective. The development of antimicrobial resistance is a major cause of therapeutic failure, posing a significant challenge to disease control [6].

Due to this point, the exploration of a therapeutic alternative that does not cause resistance has been pushed to reduce the usage of antimicrobials. Herbal medicine is one such promising avenue. Plant-derived compounds have the advantage of not inducing resistance, even after prolonged use [7]. Numerous plants have been demonstrated to possess antimicrobial properties. A study by Kimestri [8] stated that *Caesalpinia sappan* (Sappan wood) has demonstrated effectiveness against various pathogenic bacteria, including *E. coli*, *S. aureus*, *Shigella flexneri*, *Salmonella Typhimurium*, and *Listeria monocytogenes*. These bacteria are known pathogens that can cause a range of infections in both animals and humans. Additionally, *Aloe vera* has been used for wound healing by increasing tissue formation and reducing inflammation [9]. Given the therapeutic properties of both herbs, they represent potential for use in mastitis treatment. The objectives of this study were to investigate the bacterial species associated with bovine mastitis, assess their antimicrobial resistance profiles, evaluate the in vitro activity of *C. sappan* extracts against these pathogens, and develop an intramammary infusion of *C. sappan* and Aloe vera-based extract compound as a prototype for mastitis treatment. Understanding the bacterial species involved, their resistance characteristics, and exploring new therapeutic alternatives could contribute to reducing the incidence of mastitis in dairy herds, particularly in specific geographical regions.

## 2. Materials and Methods

### 2.1. Sample Collection

This study focused on Holstein Friesian dairy cows raised in the Maejo cooperative group, located in Chiang Mai, Thailand. The cooperative includes several farms with a total of 978 milking cows, collectively producing 318,572.62 kg of milk/month, with an average milk yield of 10.85 kg/cow/day.

**Figure 1:**
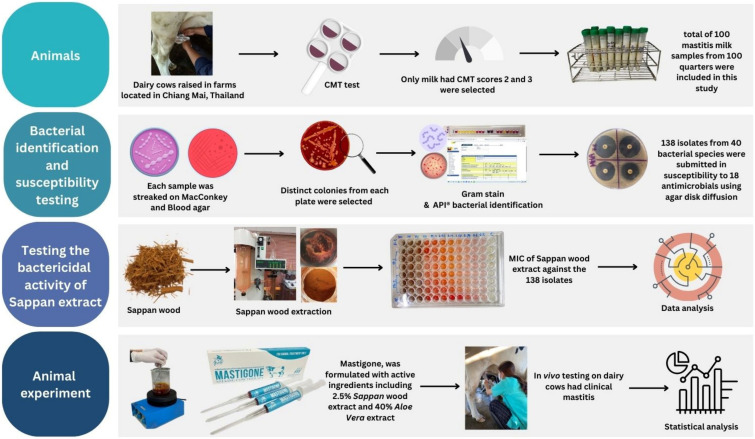
A schematic diagram summarizing the entire workflow of the study.

All procedures involving the experimental cows were conducted under ethical approval reference number MACUC014A/2563 from the Maejo University Animal Care and Use Committee. The study was conducted during the period from May 2021 to February 2022. A schematic diagram summarizing the entire workflow of the study is presented in Figure 1. The udders of the cows were washed with clean water, wiped with a towel, and the teats were surface disinfected using a pre-dipping solution. After discarding the first stream of milk, the California Mastitis Test (CMT) was performed.

A total of 100 milk samples from 100 quarters (40–50 mL each) that exhibited CMT scores of 2 or 3 (Table 1 [10]) were included in the experiment. The sample size was calculated using the WinEpi online program (http://www.winepi.net/uk/index.htm, accessed on 25 May 2021) [11]. The expected prevalence rate, representing the probability of bacterial presence in milk samples testing positive with the CMT, was 94.95% [12]. An accepted error rate of 5% and a 95% confidence level were selected as the required input parameters. For an infinite population, a minimum of 74 milk samples was determined. However, to enhance accuracy and reliability, additional samples were carefully selected. Each milk sample was collected in a sterile, separately packaged tube and transported in an icebox to the Bacteriology Laboratory Section, Faculty of Animal Science and Technology, Maejo University, for processing within 24 h.

### 2.2. Microbial Identification and Susceptibility Testing

Genus and species identification were performed following the method described by the study of Pașca [3]. Briefly, milk samples were streaked onto MacConkey agar and Blood agar (Oxiod, Hampshire, UK). After incubation at 37 °C for 24 h, the plates were examined for colony morphology, pigmentation, and hemolytic characteristics. Distinct colonies from each plate were selected for Gram staining. Each colony was analyzed to determine the appropriate Analytical Profile Index (API) type (bioMérieux Inc., Durham, NC, USA). Rapid identification of clinically relevant species was performed by observing positive reactions in small tubes containing nutrient substrates, with results verified against established bacterial databases. All bacterial isolates identified were counted and documented for further analysis.

The bacterial isolates were then tested for susceptibility to a panel of 18 different antimicrobials using the agar disk diffusion method, following the guidelines of the European Committee on Antimicrobial Susceptibility Testing [13]. Antimicrobials tested comprised Amikacin (AK) 30 µg, Amoxicillin–clavulanic acid (AMC) 20/10 µg, Amoxicillin (AML) 10 µg, Cefotaxime (CTX) 30 µg, Cloxacillin (OB) 5 µg, Streptomycin (S) 10 µg, Penicillin G (P) 10 µg, Lincomycin (MY) 15 µg, Gentamicin (CN) 120 µg, Neomycin (N) 30 µg, Cephalothin (KF) 30 µg, Ceftiofur (EFT) 30 µg, Cephalexin (CL) 30 μg, Ceftriaxone (CRO) 30 µg, Sulfamethoxazole (SXT) 25 µg, Ciprofloxacin (CIP) 10 µg, Oxytetracycline (OT) 30 µg, and Enrofloxacin (ENR) 5 µg. The *Escherichia coli* ATCC^®^ 25922 [14] was used as a control. Isolates were classified as resistant to a given antimicrobial if the inhibition zone diameter was smaller than the established threshold value (mm), as demonstrated in Supplementary Table S1. To prevent overestimation of resistance, all isolates exhibiting intermediate resistance were classified as susceptible.

### 2.3. Testing the Bactericidal Activity of Herbal Preparations

The naturally grown *C. sappan* was harvested, and its heartwood was selected and cleaned by removing impurities. The wood was washed thoroughly and then dried in a hot air oven at 60 °C for 24 h. After drying, it was cut into small pieces. The extraction was carried out using deionized water at a ratio of 1:10 (Sappan wood to deionized water). The mixture was then incubated in a water bath at a controlled temperature of 90 °C for 1.5 h. The resulting extract was further processed by spray drying and stored in an opaque container. Finally, the phenolic compound content was analyzed.

The agar microdilution method, modified from Golus et al. [15], was used to test all bacterial isolates, following the guidelines of the Clinical and Laboratory Standards Institute [16]. *C. sappan* extract was diluted in sterile distilled water using a two-fold serial dilution, resulting in final concentrations of 200, 100, 50, 25, 12.5, 6.25, 3.13, 1.56, 0.78, 0.4, and 0.2 mg/mL in 96-well microtiter plates. Each well contained an equal volume of 100 μL, including controls. Molten Mueller–Hinton agar (Oxiod, UK) with the volume of 50 μL/well, was added to the 96-well plates, and a 2 μL aliquot of bacterial suspension (equivalent to 10^5^ CFU/mL based on the McFarland standard) was inoculated into each well. The plates were then incubated at 37 °C for 24 h. Minimum inhibitory concentrations (MICs) were determined by visually assessing turbidity as an indicator of bacterial growth. Each test was performed in triplicate for each bacterial isolate to ensure accuracy.

### 2.4. Construction of Phylogenetic Tree and Data Annotation

A total of 41 bacterial 16S rRNA gene sequences (40 from related field isolates and 1 reference sequence from *S. aureus* 16S ribosomal RNA [16S rRNA] gene, GenBank accession: L37597.1) were retrieved from the NCBI database (https://www.ncbi.nlm.nih.gov, accessed on 30 November 2024) using their respective GenBank accession numbers. The sequences were aligned using a standard multiple sequence alignment tool to ensure accurate comparison of conserved regions. A phylogenetic tree was constructed using the FastME algorithm, a reliable distance-based method for generating phylogenies. Minimum inhibitory concentration (MIC) values were annotated onto the tree using the Interactive Tree of Life (iTOL) platform (https://itol.embl.de/, accessed on 30 November 2024) [17]. This annotation facilitated the visualization of relationships between bacterial taxa and their corresponding MIC values, providing insights into the antimicrobial activity of the tested extract across phylogenetically diverse organisms.

### 2.5. Animal Experiment

The prototype herbal intramammary infusion was formulated as a therapeutic treatment for mastitis in dairy cows. Its active ingredients included *C. sappan* extract at the minimum inhibitory concentration effective against all tested bacterial species, combined with 40% Aloe vera extract. The Aloe vera extract was sourced from Thai-China Flavors and Fragrances Industry Co., Ltd. (Phra Nakhon Si Ayutthaya, Thailand).

The prototype product was tested in 14 individual quarters with CMT scores of 3. The sample size was determined using G * Power (effect size = 0.85, α error probability = 0.05, power [1-β error probability] = 0.8). The study was conducted on five volunteer dairy farms that expressed interest in evaluating the efficacy of the product. The 14 selected quarters received an intramammary infusion of 10 mL of the product once daily for three consecutive days. Milk samples were aseptically collected before treatment and 24 h after the final treatment to compare total bacterial count and CMT scores.

### 2.6. Statistical Analysis

Statistical analysis was performed using R Studio^®^ version 2024.09.0+375. Descriptive statistics were used to calculate the frequency of bacterial species identified and the geometric mean of the MIC for the *C. sappan* extract. In the animal experiment, the log_10_ total bacterial count and CMT scores in milk were analyzed using the matched pairs sample *t*-test and Wilcoxon signed-rank test, respectively.

## 3. Results

**Figure 2:**
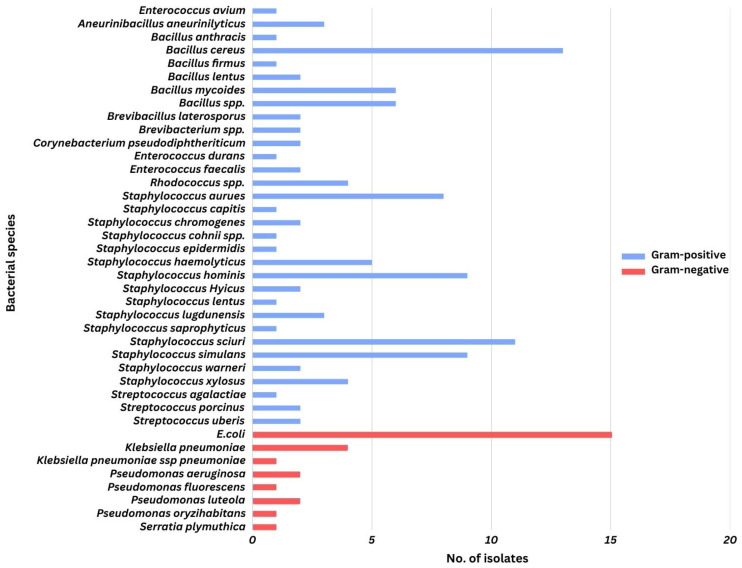
The distribution of bacterial species isolated from the subclinical mastitis milk in the study.

A total of 100 milk samples were analyzed, yielding 138 bacterial isolates representing 40 different species associated with subclinical/clinical mastitis. Of these 100 samples, 98 were bacteriologically positive. The distribution of bacterial species per sample was as follows: no bacterial growth was detected in 2 samples, a single bacterial species was identified in 62 samples, two bacterial species were in 32 samples, and three bacterial species were in 4 samples. Among the isolates, most of them were Gram-positive (111 isolates; 80.43%). Bacteria belonging to the genera *Staphylococcus* and *Streptococcus* were predominant. However, the most prevalent species was *E. coli*, accounting for 15 isolates (10.87%), followed by *B. cereus* (13 isolates; 9.42%), *S. sciuri* (11 isolates; 7.97%), *S. simulans* (9 isolates; 6.52%), and *S. hominis* (9 isolates; 6.52%) (Figure 2).

**Figure 3:**
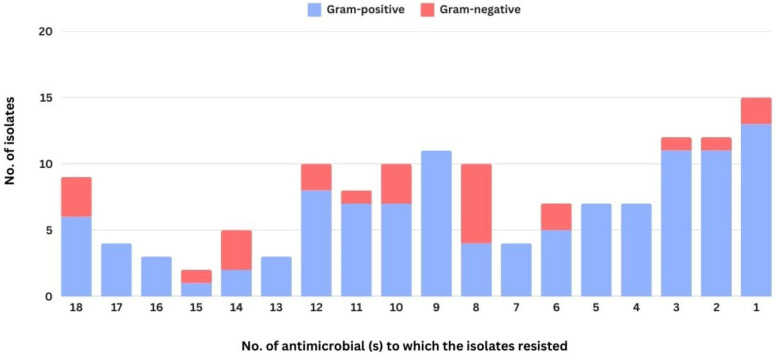
The distribution of the number of antimicrobial agents to which the bacterial isolates resisted in this study.

**Figure 4:**
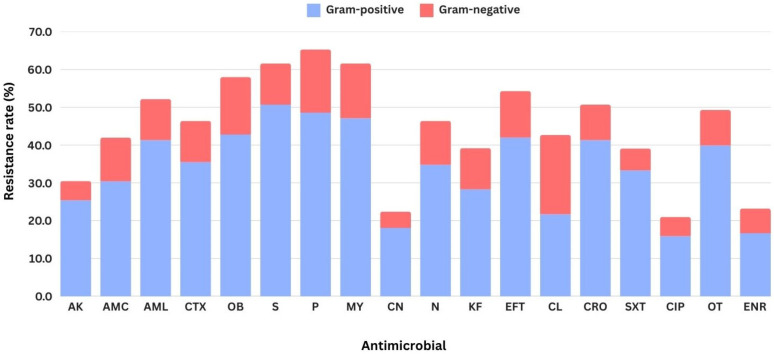
The resistance rates against 18 antimicrobials of 138 bacterial isolates originated from subclinical mastitis milk in the study. Note: abbreviations of the antimicrobials tested: Amikacin (AK), Amoxicillin-clavulanic acid (AMC), Amoxicillin (AML), Cefotaxime (CTX), Cloxacillin (OB), Streptomycin (S), Penicillin G (P), Lincomycin (MY), Gentamicin (CN), Neomycin (N), cephalothin (KF), Ceftiofur (EFT), Cephalexin (CL), Ceftriaxone (CRO), Sulfamethoxazole (SXT), Ciprofloxacin (CIP), Oxytetracycline (OT), and Enrofloxacin (ENR).

Antimicrobial susceptibility testing revealed that most bacterial isolates exhibited multidrug resistant, with 113 out of 138 isolates being resistant against at least three antimicrobial agents tested. The distribution of them and the others was demonstrated in Figure 3. Overall, the resistance was highest to penicillin (90 isolates; 65.21%), followed by streptomycin and lincomycin (85 isolates; 61.59%). In contrast, the lowest prevalence of resistance was observed for ciprofloxacin (28 isolates; 20.29%), followed by enrofloxacin (31 isolates; 22.46%) (Figure 4). In general, the antimicrobial resistance rate demonstrated in the stack bar chart for Gram-negative bacteria appears to be lower. However, this is not surprising, as Gram-negative bacteria accounted for only one-fifth of the total isolates in this study. Except for cephalexin, which exhibited a similar proportion of resistance. A total of 114 distinct resistance patterns were identified (Supplementary Table S2). Notably, the most prevalent pattern was “pan-drug resistance”, the resistance against all 18 antimicrobials tested (AK-AMC-AML-CTX-OB-S-P-MY-CN-N-KF-EFT-CL-CRO-SXT-CIP-OT-ENR), which was detected in nine isolates (6.52%), including three isolates of *B. cereus*, two isolates of *E. coli*, and each isolate of *B. mycoides*, *B. laterosporus*, *C. pseudodiphtheriticum* and *K. pneumoniae*.

**Figure 5:**
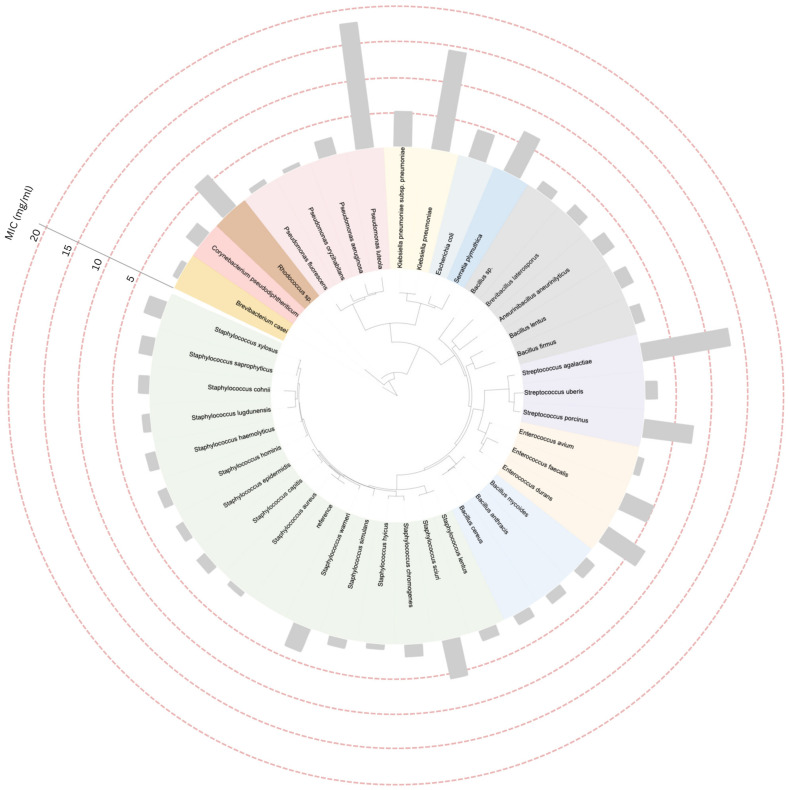
The phylogenetic trees of mastitis-causing bacteria at a species level generated using the 16S rRNA gene with MIC data against the *C. sappan* extract. Note: the similar background color of the labels represents the similar genus members.

Composition analysis revealed that the *C. sappan* crude extract contained 0.61% phenolic compounds; the MICs against all bacterial isolates were then tested. A phylogenetic tree based on 16S rRNA sequences obtained from the NCBI taxonomy database, incorporating minimum inhibitory concentration (MIC) data, was constructed using the Interactive Tree of Life (iTOL) platform. The *S. aureus* strain MRSA (16S ribosomal RNA gene, partial sequence; GenBank accession: OR527118.1) served as the reference strain. Taxa belonging to the same genus are represented by the same color on the trees. The overall average MIC values of the *C. sappan* extract against various isolated mastitis-causing bacterial species ranged from 0.63 to 17.68 mg/mL, with individual MIC values ranging from 0.4 to 25 mg/mL. The highest average MIC value was observed in *P. luteola* (17.68 mg/mL), followed by *K. pneumoniae* (14.03 mg/mL) and *S. agalactiae* (12.5 mg/mL). In contrast, the lowest MIC values were recorded for *P. oryzihabitans* (0.63 mg/mL), *P. fluorescens* (0.79 mg/mL), and *S. hyicus* (0.79 mg/mL). In general, the MIC value ranges and the arrangement of bacterial species on the phylogenetic tree appeared to be unrelated, except for the genera of *Staphylococcus* and *Bacillus*. The MIC values were predominantly lower than 5 mg/mL (Figure 5).

In the animal experiment, 14 selected quarters were targeted. A concentration of 25 mg/mL (2.5%) of the *C. sappan* extract was used. Treatment with the prototype pharmaceutical intramammary infusion for three consecutive days significantly reduced the mean total bacterial count from 10^6.9^ to 10^2.7^ CFU/mL (*p* < 0.01) and decreased the milk CMT score by two levels (Table 2).

## 4. Discussion

This study targeted dairy cows raised within a single cooperative group. The average milk yield across all member farms was approximately 11 kg/cow/day, which was quite lower than the national average daily milk yield in Thailand which was reported to be 12.17 kg/cow/day [18]. Some subclinical issues appear to be present in the selected herds, with mastitis inferred as a major factor contributing to reduced milk production and potentially impacting economic returns [19,20].

Out of one hundred CMT-positive milk samples tested in the study, only two samples did not yield detectable bacteria based on the principle of the CMT, which relies on the test reagent reacting with DNA and white blood cells in milk to form a gel [21]. However, elevated somatic cell counts in milk which potentially arise from physiological changes, such as acute dry-off periods, late lactation, stress, or systemic illnesses affecting the animal, may result in false-positives [22,23]. On the other hand, more than one-third of all samples yielded 2–3 bacterial species. Predictably, this is often observed in natural infections. Under the circumstances, multiple infections increase the likelihood of antimicrobial resistance, reduce the effectiveness of single-antimicrobial treatments, and promote the long-term survival of pathogens [24].

Infections caused by *S. aureus* and *S. agalactiae*, known pathogens of contagious mastitis [25,26], accounted for approximately 6.5% of all species detected in our study. While considered rare, these pathogens cannot be overlooked, as they can persist and proliferate within the mammary gland, facilitating the transmission from infected to uninfected quarters and between cows during the milking process [27]. Preventing contagious mastitis requires strict adherence to proper sanitation practices, such as using individual towels for each cow and implementing post-milking dipping to minimize the transmission risk [28]. Nonetheless, more than four-fifths of the bacterial species detected in this study were identified as causative agents of environmental mastitis, with *E. coli* being the most prevalent. Other species, including various *Staphylococcus* and *Streptococcus* species (which were predominant in our study) along with several additional bacterial species, were also classified within this group. These pathogens are often associated with cows frequently lying on grass plots or in environments with inadequate sanitation. Since most environmental mastitis pathogens are part of the normal fecal flora of dairy cows, providing cows with clean and dry resting areas is crucial to preventing the introduction of these pathogens into their udders. Additionally, the use of sanitized equipment and proper pre-milking dipping practices can significantly reduce the risk of pathogen transmission [29].

Monitoring antimicrobial resistance in bacteria causing mastitis holds significant clinical and public health importance, as antimicrobial therapy is commonly employed for the prevention and control of mastitis. Unfortunately, despite the use of optimal antimicrobial treatments, failures in achieving bacteriological cures are frequent, and improperly processed milk contaminated with drug-resistant pathogens can serve as a carrier posing a risk to human health [5,30,31]. In our study, the highest levels of resistance were observed against penicillin, followed by streptomycin and lincomycin. Penicillin and streptomycin are broad-spectrum agents commonly used for the treatment and prevention of diseases caused by a variety of Gram-positive and Gram-negative bacteria [5]. Both antimicrobials are considered first-line options, even in the absence of bacterial culture results or drug susceptibility data. These findings highlight the urgent need to implement strategies that encourage the prudent and responsible use of antimicrobials [31]. Although lincomycin is not widely used in the study region or in Thailand, resistance to this drug was found in various bacterial species. Indicating that no clear pattern or trend can be identified to explain the cause of drug resistance. This presents an opportunity for further research to gain a deeper understanding of the underlying factors. Interestingly, the use of high-generation antimicrobials, such as third-generation cephalosporins like cefotaxime and ceftiofur, raises further alarms. These antimicrobials are classified as critically important by the World Health Organization and are strictly regulated for use in food-producing animals due to the risk of transmitting resistant bacteria to humans and the potential failure of therapeutic treatments [30]. Therefore, this also confirms that the rational and appropriate use of antimicrobials, coupled with stringent control measures, is essential to mitigate these risks [31]. Fluoroquinolones were the most effective antimicrobial agents against spontaneous mastitis-infected bacteria in our study, with resistance rates to ciprofloxacin and enrofloxacin found to be 20% and 22%, respectively. These findings support the beneficial effects of fluoroquinolones in treating mastitis, particularly when caused by Gram-negative bacteria [32]. Therefore, identifying the type of pathogen before initiating treatment is considered crucial.

Most of the bacterial isolates identified in this study were multidrug-resistant, nine of them were classified as pan-drug resistant, meaning that they exhibited resistance to all antimicrobials tested. Based on our literature review, studies conducted approximately five years ago in this region, including southern Thailand [33], Pakistan [34], China [35], and Bangladesh [36], did not report any cases of pan-drug resistance. At least one antimicrobial remained effective against specific mastitis pathogens, particularly fluoroquinolones and aminoglycosides [33,34]. Another contributing factor is the differences in the antimicrobial panels used across studies, which have led to variations in the reported results. Higher-generation antimicrobials, such as cefquinome [35] and meropenem [36], were still 100% effective against the mastitis pathogens tested. However, our study highlights the growing severity of these pathogens, making infections more difficult to treat and potentially leading to increased morbidity, mortality, and economic losses [5]. Multidrug-resistant pathogens are highly prevalent and are recognized as a significant concern in mastitis cases on dairy farms worldwide [25,28,31,33,34,35,36]. The transference of resistance genes to other pathogenic and non-pathogenic microorganisms within farm environments, as well as between hosts, can occur readily [37], and numerous studies have demonstrated a close correlation between the development of multidrug-resistant bacteria in animals and those found in human patients, as exemplified by the “One Health” concept [5,6,30,38].

The average MIC values of the *C. sappan* crude extract against all bacterial isolates ranged from 0.63 to 17.68 mg/mL, with four-fifths of the detected species showing MIC values of 5 mg/mL or lower. This suggests that *C. sappan* possesses the ability to inhibit various multidrug-resistant bacterial strains associated with field mastitis. Srinivasan et al. [39] reported that phenolic components, such as dibenzoxocins, flavones, homoisoflavonoids, chalcones, xanthones, and brazilin, have been reported to exert antioxidant, anti-inflammatory, and antibacterial activities. However, in our study, MIC values exceeding 10 mg/mL were observed in three bacterial species, *P. luteola*, *K. pneumoniae*, and *S. agalactiae*, reflecting a relatively lower efficacy against these strains. Interestingly, a study by Nirmal and Panichayupakaranant [40] reported that brazilin-rich *C. sappan* extracts exhibited high efficacy against similar bacteria, with MIC values ranging from 15.6 to 1000 µg/mL, which were notably lower compared to our findings. Brazilin is the primary bioactive compound in the extract. Its mechanism of action is believed to involve the inhibition of bacterial DNA and protein synthesis [39]. Additionally, this compound plays a key role in modulating the immune response by binding to and inhibiting pro-inflammatory cytokines while enhancing macrophage activation [41]. Therefore, differences in extraction methods, active ingredient concentrations, plant sources, and various strain-specific traits or intrinsic microbial factors are likely contributing factors [42,43]. This presents an opportunity for further research to better understand the mechanisms of action of specific bioactive compounds in relation to their targets.

In the phylogenetic tree analysis, the genera *Staphylococcus* and *Bacillus* predominantly exhibited MIC values below 5 mg/mL, which tended to correlate with other closely related Gram-positive species. In contrast, Gram-negative bacteria, especially *P. luteola* and *K. pneumoniae*, exhibited significantly higher MIC levels. Are Gram-negative species generally more resistant to *C. sappan*? Previous studies have indicated that *C. sappan* can inhibit both Gram-positive and Gram-negative bacteria [8,36], but it appears to be less effective against Gram-negative bacteria [37]. In general, Gram-negative bacteria exhibit greater resistance to antimicrobial agents than Gram-positive bacteria due to differences in cell wall structure. The cell wall of Gram-positive bacteria, which has a simpler structure, is more permeable to various antibiotics compared to the Gram-negative cell wall, which is organized similarly to other biological membranes [44]. It is important to acknowledge the limitations of this study, as it does not universally represent all bacterial strains. Expanding the dataset to include additional field strains would improve the robustness and generalizability of the findings, providing more comprehensive knowledge.

As part of the development of an intramammary infusion pharmaceutical prototype directed at field practice, Aloe vera extract was selected for combination with *C. sappan* due to its therapeutic effects of suppressing inflammatory mediators and enhancing tissue formation [45]. The results of the animal experiment met expectations. The prototype significantly reduced bacterial counts and lowered the milk CMT score, suggesting that the combination of these two herbal extracts exhibits antimicrobial efficacy against mastitis-causing bacteria. Although the sample size of 14 quarters may seem limited, a retrospective power compute yielded a value greater than 0.99, indicating a high probability of detecting a true effect [46]. These findings confirm that this approach holds promise as an alternative treatment for managing subclinical/clinical mastitis in dairy cattle.

## 5. Conclusions

Herbal medicine is an alternative approach to replacing antimicrobial use in mastitis treatment. Our study demonstrates that *C. sappan* exhibits therapeutic potential due to its antimicrobial properties against various antimicrobial-resistant mastitis-causing pathogens, as evidenced by both in vitro and in vivo studies. However, the success of on-field mastitis treatment depends on multiple factors, including improving farm sanitation, enhancing milking hygiene, using pre- and post-milking teat dipping, and maintaining milking machines. Moreover, an effective mastitis control program should emphasize prevention over treatment. The early detection of new cases is essential to reduce costs, minimize production losses, and improve cure rates. For future studies, the economic returns and cost-effectiveness of *C. sappan* implementation should be evaluated and compared with other conventional approaches to gain a deeper understanding and enhance the safety and sustainability of farming development in the region.

## Figures and Tables

**Table 1:** The CMT score levels for subclinical–clinical mastitis evaluation.

Milk Quality	Reaction	Characteristics of Reaction
Normal, Very Good	0	A homogeneous mixture with a pale purple color that moves quickly when the CMT paddle is rotated.
Normal, Good	T	The mixture becomes mucus-like, forms a thread, and then disappears. It exhibits a pale purple color and moves quickly when the CMT paddle is rotated.
Normal, Fair	1	The mixture is viscous and mucus-like, with the purple color intensifying and moving more slowly when the CMT paddle is rotated.
Subclinical Mastitis	2	The mixture is viscous and mucus-like, with the purple color intensifying. It moves very slowly when the CMT paddle is rotated, and the udders still appear visually normal.
Clinical Mastitis	3	The mixture is thick and mucus-like, making it easily observable.

Note: the table is adapted from Philpot and Nickerson [10].

**Table 2:** The treatment results (mean ±) of infected quarters (n = 14) with the 2.5% *C. sappan* and 40% *Aloe vera* extract intramammary infusion compound.

Parameters	Before	After	*p*-Value
Total bacterial count (log_10_CFU/mL)	6.875 ± 1.72	2.704 ± 2.91	<0.01
CMT score	3 ± 0	1 ± 0	<0.01

Note: mean ± sd for total bacteria count; median ± IQR for CMT score.

## Data Availability

No new data was created in this study. The datasets used and/or analyzed during the current study are available from the corresponding author on reasonable request.

## Supplementary Materials

The following supporting information can be downloaded at https://www.mdpi.com/article/10.3390/biology14040350/s1: Table S1: The inhibition zone diameter threshold value (mm) assigned for resistance against antimicrobials tested on mastitis pathogens; Table S2: Antimicrobial resistance patterns of all 138 mastitis pathogens identified in this study.
